# Multisensory modulation of body ownership in mice

**DOI:** 10.1093/nc/niz019

**Published:** 2020-01-23

**Authors:** Christine L Buckmaster, Julia E Rathmann-Bloch, Luis de Lecea, Alan F Schatzberg, David M Lyons

**Affiliations:** Department of Psychiatry and Behavioral Sciences, Stanford University, Palo Alto, CA, USA

**Keywords:** body ownership illusions, multisensory perception, visuotactile integration, affective touch, sex differences, comparative biology

## Abstract

Body ownership is a fundamental aspect of self-consciousness that reflects more than the presence of physical body parts. As demonstrated by the rubber hand illusion (RHI), human brains construct body ownership experiences using available multisensory information. Experimental conditions similar to those that induce the RHI in humans have been recently adapted to induce the rubber tail illusion (RTI) in mice. Here, we show that the RTI is enhanced in both sexes of mice by repetitive synchronous stroking comprised of correlated visual and tactile stimulation of real and rubber tails compared to visual-only mimicked stroking conducted without tactile stimulation. The RTI also appears to be enhanced in female but not male mice by slow compared to fast stroking that reflects an interoceptive manipulation associated with affective touch in humans. Sex differences in slow stroking effects are exploratory and require replication in mice. Sex differences have not been reported for the RHI in healthy humans, but women rate slow stroking as more affectively pleasant compared to the ratings of men. Results suggest that the RHI in humans resembles aspects of the RTI in mice. Studies of mice may therefore provide neurobiological insights on evolutionarily conserved mechanisms of bodily self-consciousness in humans.

**Highlights**

- Synchronous versus visual-only mimicked stroking enhances an illusion of body ownership in both sexes of mice.
- The same illusion appears to be enhanced by slow versus fast stroking in female but not male mice.
- Brains construct body ownership illusions from available multisensory information in humans and in mice.
- Studies of mice may provide insights on evolutionarily conserved aspects of bodily self-consciousness in humans.

## Introduction

Body ownership is a fundamental aspect of self-consciousness that reflects more than simply the presence of physical body parts (Seth and Tsakiris 2018). As demonstrated by the rubber hand illusion (RHI) discovered by Botvinick and Cohen (1998), human brains construct body ownership experiences using available multisensory information. In the past 20 years, RHI has become one of the most extensively studied experimental manipulations of body ownership with more than 375 published reports indexed on PUBMED.

To induce RHI, a person’s own hand is hidden from view while a visible rubber hand is placed in an anatomically plausible position with respect to the body. Hidden real and visible rubber hands are then repetitively stroked synchronously or asynchronously. Compared to repetitive asynchronous stroking, repetitive synchronous stroking results in the illusion that the rubber hand subjectively belongs to the person, and that distances between the visible rubber hand and perceived location of the hidden real hand are smaller than objective measurements demonstrate that they actually are, i.e., proprioceptive drift (Ramakonar *et al.* 2011). In addition to subjective reports and objective measures of proprioceptive drift, another objective RHI measure is real hand galvanic skin responses when the rubber hand is threatened with harm (Armel and Ramachandran 2003). Subjective and objective responses to various control treatments suggest that RHI reflects perceptual assimilation and not associative conditioning (Armel and Ramachandran 2003). Not all people experience RHI, but 30 s of repetitive synchronous stroking is generally sufficient to induce RHI in 70% of healthy humans (Costantini 2014).

Using RHI-like procedures, Wada *et al.* (2016, 2019) discovered the rubber tail illusion (RTI) in mice. Real and rubber tails are repetitively stroked synchronously or asynchronously, and then head withdrawal movement responses are assessed when the rubber tail is firmly grasped. Defensive head withdrawal responses are monitored because in human and non-human primates motoric defense of the body is an indicator of body ownership (Graziano and Cooke 2006; Ehrsson *et al.* 2007). Wada *et al.* (2016, 2019) reported that when real and rubber tails are repetitively stroked synchronously, male mice subsequently respond as if their own tail is grasped when the rubber tail is pinched. In contrast, when real and rubber tails are repetitively stroked asynchronously, male mice demonstrate diminished RTI behavioral responses (Wada *et al.* 2016; Wada *et al.* 2019). RTI responses in male mice are also diminished after repetitive synchronous stroking of real and rubber tails when the rubber tail is occluded from view by an opaque barrier (Wada *et al.* 2016). Results suggest that integration of correlated visual and tactile stimulation is important for RTI in mice as has been hypothesized for RHI in humans (Botvinick and Cohen 1998; Armel and Ramachandran 2003; Kilteni *et al.* 2015; Guterstam *et al.* 2019).

Here, we further investigate RTI as evidence of body ownership in mice. First, we test the hypothesis that repetitive synchronous stroking comprised of correlated visual and tactile stimulation of real and rubber tails enhances RTI behavioral responses compared to visual-only mimicked stroking conducted without tactile stimulation in both sexes of mice. Evidence suggests that visual-only stimulation may or may not be sufficient for induction of RHI in humans (Durgin *et al.* 2007; Rohde *et al.* 2011; Ferri *et al.* 2013; Guterstam *et al.* 2019), and we investigate this issue for RTI in mice. Then, we test the hypothesis that RTI behavioral responses in mice are enhanced by slow compared to fast stroking treatments that represent an interoceptive manipulation associated with affective touch in humans (Crucianelli *et al.* 2013; Lloyd *et al.* 2013; van Stralen *et al.* 2014; Crucianelli *et al.* 2018).

Affective touch is thought to be mediated by C-tactile afferents that project from hairy but not glabrous skin (Crucianelli *et al.* 2018). Mouse tails are comprised of hairy skin (Duverger and Morasso 2009), and the mouse equivalent of C-tactile afferents are called C-low threshold mechanoreceptors (C-LTMRs), which reside in mouse hairy skin (Liu *et al.* 2007; Li *et al.* 2011). Like C-tactile afferents in humans, C-LTMRs in mice selectively respond to slow stroking (Vrontou *et al.* 2013). Experimental evidence further suggests that C-LTMRs causally mediate positively reinforcing behavioral effects of slow stroking in mice (Vrontou *et al.* 2013). Therefore, we test for RTI modulation by slow compared to fast stroking treatments in both sexes of mice.

## Materials and Methods

Subjects included eight male and eight female C57BL/6J mice purchased as adults from Charles River (Gilroy, CA). Mice were housed in groups of four same-sex individuals in climate-controlled rooms with an ambient temperature of 26°C. Food and water were provided *ad libitum*. Treatment and test procedures were conducted during the light phase of the circadian cycle with lights on from 07:00 to 19:00 h. All procedures were conducted in accordance with state and federal laws, standards of the US Department of Health and Human Services, and the Institutional Animal Care and Use Committee at Stanford University.

**Figure 1.**
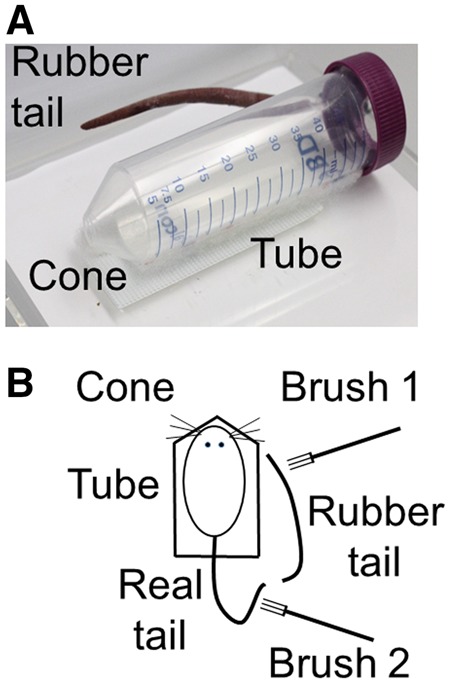
RTI apparatus. Photograph (A) and schematic representation (B).

Each mouse was individually acclimated to standing stationary while loosely restrained in a clear plastic 50 ml BD Falcon tube tapered to a cone at one end (Fig. 1). The other end of the tube was partially enclosed to accommodate the tail through a hole in the screw-on cap. Head and body movements forward and backward were possible, but mice could not turn around in the tube. Acclimation was accomplished when each mouse stood stationary in the tube for ≥45 of 60 s (75%) on each of three consecutive daily assessments.

The tube was fixed onto a 15 cm × 15 cm plastic platform raised 10 cm above bench top with the rubber tail attached to the platform on the right side of the tube. Because mice can bend their tails forward, the rubber tail was located in a plausible anatomical position. Human RHI research indicates that improbable positioning causes a breakdown of the illusion (Lloyd 2007; Brozzoli *et al.* 2012). To prepare the rubber tail, a matte colored photograph of a real tail was adhered to heavy paper 9.5 cm in length and graduated from 5 mm at the base to 2 mm at the tip of the tail. In various stroking treatments described below, real and rubber tails were manually stroked repetitively for 1-min using separate Filbert flat art brushes with synthetic soft bristles (10 mm width by 20 mm length). The real tail was always hidden from view while the rubber tail was visible to the mouse.

First, we used a within-subjects counterbalanced cross-over design to compare synchronous and visual-only mimicked stroking treatment conditions in both sexes of mice. Each condition was applied for a 1-min trial per day for 5 days per condition. In the synchronous condition, real and rubber tails were repetitively stroked synchronously for 1-min with correlated visual and tactile stimulation. In the visual-only mimicked condition, repetitive synchronous stroking was mimicked for 1-min with sweeping motions conducted 1 cm above the real and rubber tails without ever touching either tail. The latter condition is similar to treatments used by Guterstam *et al.* (2019) in RHI studies of humans and represents an alternative to the asynchronous control for RTI used by Wada *et al.* (2016) in male mice.

Repetitive rates of stroking applied by Wada *et al.* (2016) to male mice ranged from 0.5 to 2 Hz. In Experiment 1 described above, we examined the low end of this range in both sexes of mice and applied irregular stroking repetition rates of 0.5–0.9 Hz at a constant velocity of 3 cm/s along a 3 cm length of tail. Velocities have been systematically compared in RHI studies of humans, but repetition rates were either varied irregularly, concurrently with velocities, or were not specified (Crucianelli *et al.* 2013; Lloyd *et al.* 2013; van Stralen *et al.* 2014; Crucianelli *et al.* 2018). Varied repetition rates have been examined in RTI studies of mice (Wada *et al.* 2016; Wada *et al.* 2019), but varied velocities have not been considered.

In two RTI experiments, we systematically varied repetition rates while holding velocity constant, or we concurrently varied repetition rates and velocities. Specifically, Experiment 2 compared a slow repetition rate of 0.1 Hz and a faster repetition rate of 0.3 Hz with both rates applied at a constant velocity of 3 cm/s along a 3 cm length of tail. Experiment 3 compared a slow velocity of 1 cm/s applied at a rate of 0.3 Hz and a faster velocity of 6 cm/s applied at a rate of 2.0 Hz along a 3 cm length of tail. Each speed-of-stroking treatment condition was applied for a 1-min trial per day for 2 days per condition using within-subjects counterbalanced cross-over designs.

Experiments were conducted sequentially using all mice with 2–3 week intervals between each of three experiments. Following Wada *et al.* (2019), a digital stopwatch was used to control delivery of the stroking treatments. All stroking treatments were practiced before each experiment on the rubber tail in the absence of a mouse by a trained investigator (C.L.B.).

Immediately after each stroking treatment condition, three rubber tail pinch tests were conducted sequentially at 3- to 5-s intervals to robustly sample head movement responses induced by the experimenter strongly grasping the rubber tail with a strength similar to that used when lifting a mouse. Because mice were loosely restrained in the RTI apparatus without holding their head fixed facing towards the rubber tail, three sequential pinch tests were conducted to robustly sample RTI pinch test behavior described below. Human RHI research indicates that illusions induced by repetitive synchronous stroking last for ∼90 s (Lane *et al.* 2017).

Digital video recordings of all pinch tests were coded for identification and scored offline as described by Wada *et al.* (2016). Briefly, if the head turned toward or away from the rubber tail, or the head or body moved forward or retracted backward into the tube, this was considered a full response and was scored as 1. If the response was partial or incomplete, this was scored as 0.5. Postural adjustment or no head movement was scored as 0.

Pinch test video recordings were independently scored by two trained raters. Video recorder failure resulted in missing data from one female mouse. One rater (J.E.R.-B.) had no knowledge of stroking treatments used in the studies, and the other rater (C.L.B.) had limited knowledge from administering all stroking treatments 3- to 14-days before scoring video recordings. From the three sequential pinch tests per mouse that followed each stroking treatment, we separately computed mean RTI pinch test response scores for each rater.

After all pinch tests were scored and inconsistencies identified for rater retraining, both raters independently rescored 90 randomly selected sets of three sequential pinch tests. From rescored data, we computed mean RTI pinch test response scores separately for each rater to evaluate inter- and intra-rater reliabilities expressed as intraclass correlation coefficients (ICC) (Koo and Li 2016). Inter-rater ICC derived from a two-way random-effects single measure model was 0.90, and intra-rater ICC derived from a two-way mixed-effects single measure model was 0.92. Because of similar inter- and intra-rater reliabilities, initially collected mean RTI pinch test response scores from each rater were averaged to serve as our unit of analysis. This approach follows Wada *et al.* (2016).

Response score distributions were assessed for normality with Kolmogorov–Smirnov tests and evaluated by analysis of variance (ANOVA). For the first experiment, stroking treatments (synchronous versus mimicked) and test days (1–5) were considered within-subjects factors, and sex was considered a between-subjects factor. The two speed-of-stroking experiments were analyzed with separate ANOVAs with stroking speed (slow versus fast) and test days (1–2) considered within-subjects factors, and sex was considered a between-subjects factor. Sex differences were also assessed using the χ^2^ test. Test statistics were evaluated at *P* < 0.05 (two-tailed), effect size estimates were determined using partial eta squared η^2^ (Fritz *et al.* 2012), and descriptive statistics were presented as mean ± SEM. Data are provided in Supplementary Tables S1–S3.

## Results

Acclimation to standing stationary in the RTI apparatus was accomplished in 4 weeks by all mice. RTI pinch test response score distributions did not differ significantly from normal in the synchronous (*P* = 0.66), mimicked (*P* = 0.98), or speed-of-stroking (*P* = 0.65) treatment conditions. Therefore, we used fully factorial ANOVAs to assess stroking treatments, test days, and sex effects.

**Figure 2.**
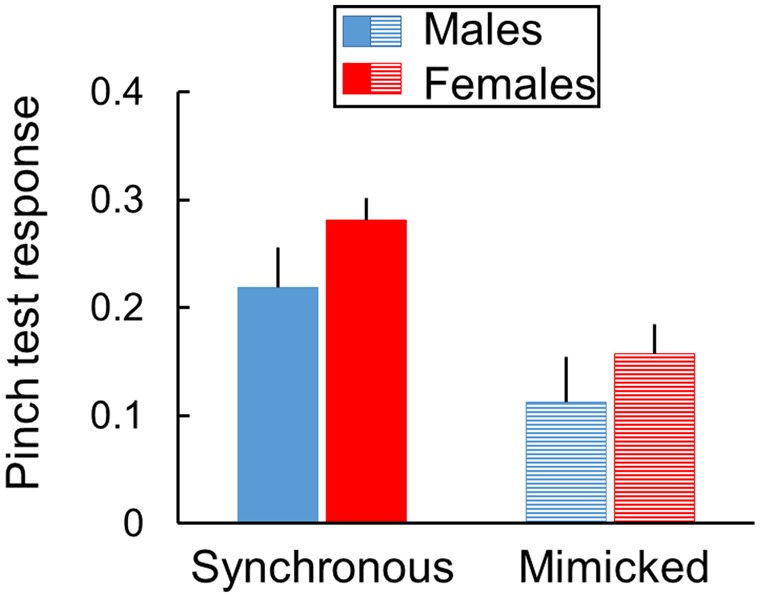
Similar synchronous versus mimicked stroking effects in both sexes of mice. Rubber tail pinch test responses are enhanced by repetitive synchronous stroking comprised of correlated visual and tactile stimulation of real and rubber tails compared to visual-only mimicked stroking conducted without tactile stimulation in both sexes of mice. The stroking treatment main effect is significant (*P* < 0.001), but the sex main effect and interaction with stroking treatment are not significant (Supplementary Table S4). Responses are averaged over 2 raters for 3 pinch tests per day and 5 days per mouse in each treatment (15 pinch tests per mouse, mean ± SEM, *n* = 7 females and 8 males).

**Figure 3.**
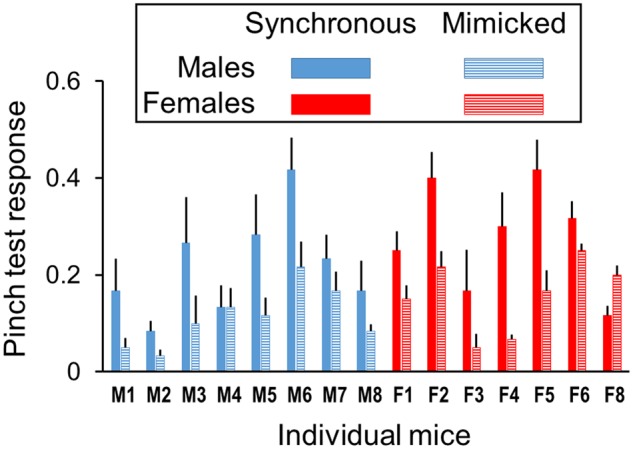
Analysis of individual mice. Rubber tail pinch test responses are enhanced in 14 of 15 mice by repetitive synchronous stroking comprised of correlated visual and tactile stimulation of real and rubber tails compared to visual-only mimicked stroking conducted without tactile stimulation. Responses are averaged over 2 raters for 3 pinch tests per day and 5 days per mouse in each stroking treatment [15 pinch tests per mouse, mean ± SEM, *n* = 7 females (red) and 8 males (blue)].

RTI pinch test responses were enhanced after synchronous compared to mimicked stroking treatments as discerned by a treatment main effect [F(1, 13) = 23.2, *P* < 0.001, partial η^2^ = 0.64]. Treatment effect sizes for males (partial η^2^ = 0.73) and females (partial η^2^ = 0.59) were similar, and statistically significant sex differences were not discerned (Fig. 2). The test day main effect was not significant (Supplementary Table S4), but a significant test day-by-sex interaction [F(4, 52) = 2.8, *P* = 0.035] was found. In both synchronous and mimicked stroking treatments, pinch test responses declined after the 4th test day in females [F(4, 24) = 4.3, *P* = 0.009], but not in males (*P* = 0.742). Pinch test responses averaged across all 5 test days for each individual mouse revealed consistent mean differences in favor of synchronous compared to visual-only mimicked stroking for 14 of 15 mice (Fig. 3).

**Figure 4.**
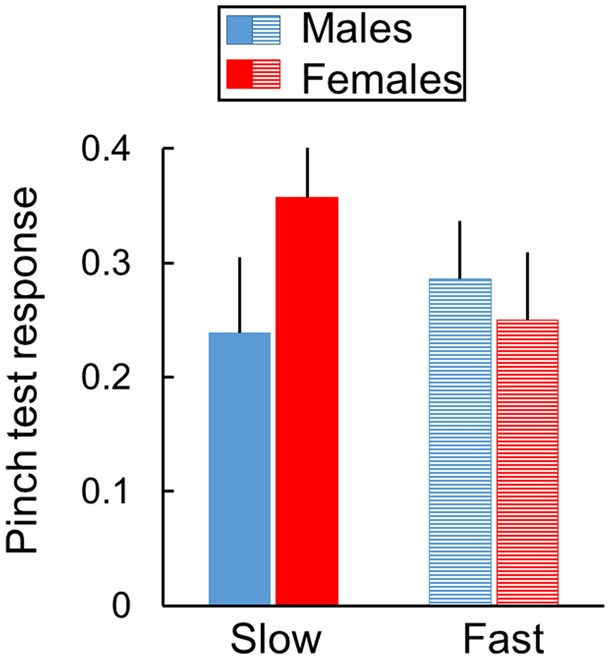
Modulation by stroking speed. Rubber tail pinch test responses are enhanced by slow compared to fast stroking in female but not in male mice when both velocities and repetition rates are varied. The stroking speed-by-sex interaction is significant (*P* = 0.029), but test day effects are not significant (Supplementary Table S5). Responses are averaged over 2 raters for 3 pinch tests per day and 2 test days per mouse in each stroking treatment (mean ± SEM, *n* = 7 females and 8 males).

Next, we systematically manipulated stroking speeds. The only significant finding was a speed-of-stroking-by-sex interaction [F(1, 13) = 6.0, *P* = 0.029, partial η^2^ = 0.32] evident only when stroking velocities and repetition rates were varied concurrently (Supplementary Table S5). For the speed-of-stroking-by-sex interaction depicted in Fig. 4, subsequent exploratory analysis revealed that RTI pinch test responses were enhanced by slow compared to fast stroking in females [F(1, 6) = 5.6, *P* = 0.05, partial η^2^ = 0.49] but not in males [F(1, 7) = 1.2, *P* = 0.316, partial η^2^ = 0.15]. When stroking repetition rates were varied alone while holding velocity constant, none of the main or interaction effects was statistically significant (Supplementary Table S6).

Sex differences in speed-of-stroking effects were further assessed by analysis of individual mice when both stroking repetition rates and velocities were varied concurrently (Supplementary Fig. S1). Mean differences in RTI pinch test responses after slow compared to fast stroking were greater in 5 of 7 females (71%) and 1 of 8 males (12%). This sex difference is statistically significant (χ^2^ = 5.4, *P* = 0.02).

## Discussion

RTI pinch test responses are enhanced by repetitive synchronous stroking comprised of correlated visual and tactile stimulation of real and rubber tails compared to visual-only mimicked stroking conducted without tactile stimulation. Similar results in both sexes extend findings by Wada *et al.* (2016, 2019) in male mice. RTI responses also appear to be enhanced in female but not in male mice by slow compared to fast stroking treatments that represent an interoceptive manipulation associated with affective touch in humans (Crucianelli *et al.* 2013; Lloyd *et al.* 2013; van Stralen *et al.* 2014; Crucianelli *et al.* 2018). Sex differences in slow stroking effects are exploratory and require replication in mice. Sex differences have not been reported for RHI in healthy humans (Guterstam *et al.* 2019), but women rate slow stroking as more affectively pleasant compared to the ratings of men (Croy *et al.* 2014; Jonsson *et al.* 2017). Results suggest that RHI in humans resembles aspects of RTI in mice. Studies of mice may therefore provide neurobiological insights on evolutionarily conserved mechanisms of bodily self-consciousness in humans.

Multisensory contributions to body ownership are difficult to determine because separate senses are tightly bound together and not normally accessible to independent experimental manipulation. Illusions induced by manipulated stimulation provide powerful tools to investigate how brains construct body ownership using available multisensory information (Costantini 2014; Kilteni *et al.* 2015). Integration of correlated visual and tactile stimulation is important for RHI in humans (Botvinick and Cohen 1998; Armel and Ramachandran 2003; Kilteni *et al.* 2015; Guterstam *et al.* 2019), and likewise contributes significantly to RTI in mice.

Wada *et al.* (2016) first reported that repetitive synchronous stroking of real and rubber tails enhances RTI behavioral responses in male mice compared to identical stroking treatments applied when the rubber tail is occluded from view. Here, we find in both sexes of mice that repetitive synchronous stroking comprised of correlated visual and tactile stimulation of real and rubber tails enhances RTI responses compared to visual-only mimicked stroking conducted without tactile stimulation. Although visual-only treatments may or may not be sufficient for induction of RHI in humans (Durgin *et al.* 2007; Rohde *et al.* 2011; Ferri *et al.* 2013; Guterstam *et al.* 2019), visual-only mimicked stroking appears to be insufficient for induction of RTI in both sexes of mice. The possibility that this finding reflects species differences in visually induced tactile expectations remains to be explored.

Repetitive rates of stroking applied by Wada *et al.* (2016) to male mice ranged from 0.5 to 2 Hz. The low end of this range was examined in Experiment 1 with irregular stroking repetition rates of 0.5–0.9 Hz applied at a constant velocity of 3 cm/s along a 3 cm length of tail in both sexes of mice. In follow-up experiments, we either systematically varied repetition rates while holding velocity constant, or we concurrently varied repetition rates and velocities. Varied velocities significantly modulate RHI in humans (Crucianelli *et al.* 2013; Lloyd *et al.* 2013; van Stralen *et al.* 2014; Crucianelli *et al.* 2018). RTI likewise appears to be modulated in female but not in male mice when velocities and repetition rates are varied concurrently, but statistically significant modulation is not evident in mice of either sex when repetition rates are varied alone.

Until recently, neurobiological and biomedical studies of mice have focused primarily on males (Beery and Zucker 2011), and RTI sex differences are largely unexplored. Although sex differences have not been reported in RHI studies of healthy humans (Guterstam *et al.* 2019), women subjectively rate slow stroking as more affectively pleasant compared to the subjective ratings of men (Croy *et al.* 2014; Jonsson *et al.* 2017). Possible neural mechanistic explanations include sex differences in brain circuits that process speed-of-stroking stimulation, or sex steroid hormone modulation of C-tactile afferent functions. Manipulations required to experimentally investigate causal neural mechanisms are often more readily accomplished in mice than in humans, and this advantage supports development of RTI as a model of body ownership in mice.

Our results should be interpreted in the context of potential limitations. Subjective experiences cannot be assessed directly in mice, but nearly identical stroking treatments can be applied to humans and mice. After various stroking treatments, we scored RTI pinch test responses with an ordinal Likert scale and converted ordinal measures into interval data by taking the mean of three sequential pinch tests per mouse after each stroking treatment. This approach follows standard recommendations (Carifio and Perla 2007), and generates data distributions that do not differ significantly from normal. Consequently, our data are suitable for parametric statistical tests (Sullivan and Artino 2013). Effect sizes are greater in mice for modulation of RTI by exteroceptive stimulation compared to interoceptive speed-of-stroking stimulation, but further studies need to vary velocities while holding repetition rate constant. Velocities have been systematically compared in RHI studies of humans (Crucianelli *et al.* 2013; Lloyd *et al.* 2013; van Stralen *et al.* 2014; Crucianelli *et al.* 2018), but repetition rates either vary irregularly, concurrently with velocities, or are not specified.

## Conclusions

RTI pinch test behavioral responses in both sexes of mice are enhanced by repetitive synchronous stroking comprised of correlated visual and tactile stimulation of real and rubber tails compared to visual-only mimicked stroking conducted without tactile stimulation. RTI responses also appear to be enhanced in female but not in male mice by slow compared to fast stroking treatments that represent an interoceptive manipulation associated with affective touch in humans. Sex differences in slow stroking effects are exploratory and require replication in mice. Sex differences have not been reported for RHI in healthy humans, but women rate slow stroking as more affectively pleasant compared to the ratings of men. Results suggest that RHI in humans resembles aspects of RTI in mice. Studies of mice may therefore provide neurobiological insights on evolutionarily conserved mechanisms of bodily self-consciousness in humans.

## Ethics

All procedures were conducted in accordance with state and federal laws, standards of the US Department of Health and Human Services, and were approved by the Institutional Animal Care and Use Committee at Stanford University.

## Data availability

Data are publically available in Supplementary Material Tables S1, S2, and S3.

## Supplementary Material

niz019_Supplementary_Data
