# Supplementary material for: Multisensory modulation of body ownership in mice
Source: Neurosci Conscious. 2020 Jan 23;2020(1):niz019. doi: 10.1093/nc/niz019 (PMC6977007; doi:10.1093/nc/niz019)
Supplement: niz019_Supplementary_Data [file niz019_supplementary_data.zip › Supplementary Table 4.docx]

**Supplementary Table S4. ANOVA for synchronous versus mimicked stroking effects.** Data presented in Supplementary Table S1 are analyzed by a fully factorial ANOVA with stroking treatments (synchronous versus mimicked) and test days considered within-subjects factors, and sex considered as a between-subjects factor. Also provided are effect size estimates η^2^.

| Source | Sum of squares | df | Mean squares | F | P value | η^2^ |
| --- | --- | --- | --- | --- | --- | --- |
| Sex | 0.107 | 1 | 0.107 | 1.779 | 0.205 | 0.12 |
| Error | 0.780 | 13 | 0.060 |  |  |  |
| Treatment | 0.495 | 1 | 0.495 | 23.184 | <0.001 | 0.64 |
| Treatment x Sex | 0.003 | 1 | 0.003 | 0.134 | 0.720 | 0.01 |
| Error | 0.277 | 13 | 0.021 |  |  |  |
| Test days | 0.191 | 4 | 0.048 | 1.550 | 0.202 | 0.11 |
| Test days x Sex | 0.347 | 4 | 0.087 | 2.814 | 0.035 | 0.18 |
| Error | 1.602 | 52 | 0.031 |  |  |  |
| Treatment x Test days | 0.151 | 4 | 0.038 | 1.029 | 0.401 | 0.07 |
| Treatment x Test days x Sex | 0.123 | 4 | 0.031 | 0.838 | 0.508 | 0.06 |
| Error | 1.914 | 52 | 0.037 |  |  |  |
