# Supplementary material for: Multisensory modulation of body ownership in mice
Source: Neurosci Conscious. 2020 Jan 23;2020(1):niz019. doi: 10.1093/nc/niz019 (PMC6977007; doi:10.1093/nc/niz019)
Supplement: niz019_Supplementary_Data [file niz019_supplementary_data.zip › Supplementary Table 5.docx]

**Supplementary Table S5. ANOVA for speed-of-stroking effects when both velocities and repetition rates are varied concurrently.** Data presented in Supplementary Table S2 are analyzed by a fully factorial ANOVA with stroking treatments (slow versus fast) and test days considered within-subjects factors, and sex considered as a between-subjects factor. Also provided are effect size estimates η^2^.

| Source | Sum of squares | df | Mean squares | F | P value | η^2^ |
| --- | --- | --- | --- | --- | --- | --- |
| Sex | 0.025 | 1 | 0.025 | 0.203 | 0.659 | 0.02 |
| Error | 1.571 | 13 | 0.121 |  |  |  |
| Treatment | 0.014 | 1 | 0.014 | 0.924 | 0.354 | 0.07 |
| Treatment x Sex | 0.089 | 1 | 0.089 | 6.019 | 0.029 | 0.32 |
| Error | 0.192 | 13 | 0.015 |  |  |  |
| Test days | 0.087 | 1 | 0.087 | 1.525 | 0.239 | 0.10 |
| Test days x Sex | 0.005 | 1 | 0.005 | 0.095 | 0.762 | 0.01 |
| Error | 0.742 | 13 | 0.057 |  |  |  |
| Treatment x Test days | 0.022 | 1 | 0.022 | 0.517 | 0.485 | 0.04 |
| Treatment x Test days x Sex | 0.016 | 1 | 0.016 | 0.385 | 0.546 | 0.03 |
| Error | 0.552 | 13 | 0.042 |  |  |  |
