# Supplementary material for: Multisensory modulation of body ownership in mice
Source: Neurosci Conscious. 2020 Jan 23;2020(1):niz019. doi: 10.1093/nc/niz019 (PMC6977007; doi:10.1093/nc/niz019)
Supplement: niz019_Supplementary_Data [file niz019_supplementary_data.zip › Supplementary Table 6.docx]

**Supplementary Table S6. ANOVA for speed-of-stroking effects when repetition rates alone are varied and velocity is held constant.** Data presented in Supplementary Table S3 are analyzed by a fully factorial ANOVA with stroking treatments (slow versus fast) and test days considered within-subjects factors, and sex considered as a between-subjects factor. Also provided are effect size estimates η^2^.

| Source | Sum of squares | df | Mean squares | F | P value | η^2^ |
| --- | --- | --- | --- | --- | --- | --- |
| Sex | 0.003 | 1 | 0.003 | 0.032 | 0.860 | 0.003 |
| Error | 1.095 | 13 | 0.084 |  |  |  |
| Treatment | 0.176 | 1 | 0.176 | 3.208 | 0.097 | 0.20 |
| Treatment x Sex | 0.032 | 1 | 0.032 | 0.582 | 0.459 | 0.04 |
| Error | 0.714 | 13 | 0.055 |  |  |  |
| Test days | 0.008 | 1 | 0.008 | 0.180 | 0.678 | 0.01 |
| Test days x Sex | <0.001 | 1 | <0.001 | 0.001 | 0.978 | 0.002 |
| Error | 0.545 | 13 | 0.042 |  |  |  |
| Treatment x Test days | 0.015 | 1 | 0.015 | 0.144 | 0.710 | 0.01 |
| Treatment x Test days x Sex | 0.015 | 1 | 0.015 | 0.145 | 0.710 | 0.01 |
| Error | 1.313 | 13 | 0.101 |  |  |  |
