# Supplementary material for: Multisensory modulation of body ownership in mice
Source: Neurosci Conscious. 2020 Jan 23;2020(1):niz019. doi: 10.1093/nc/niz019 (PMC6977007; doi:10.1093/nc/niz019)
Supplement: niz019_Supplementary_Data [file niz019_supplementary_data.zip › Supplementary Fig 1 for review.docx]

**Supplementary Figure 1. Speed-of-stroking effects in individual mice.** RTI pinch test responses are enhanced in 5 of 7 females (71%) and 1 of 8 males (12%) after slow compared to fast stroking when both stroking velocities and repetition rates are varied concurrently (χ^2^=5.4, P=0.02). Responses are averaged over 2 raters for 3 pinch tests per day and 2 days per mouse in each stroking treatment (mean of 6 pinch tests per mouse, n=7 females (red) and 8 males (blue)).
