# Supplementary material for: Multisensory modulation of body ownership in mice
Source: Neurosci Conscious. 2020 Jan 23;2020(1):niz019. doi: 10.1093/nc/niz019 (PMC6977007; doi:10.1093/nc/niz019)
Supplement: niz019_Supplementary_Data [file niz019_supplementary_data.zip › Supplementary Table 1.docx]

**Supplementary Table S1. Synchronous versus mimicked stroking data.** Rubber tail pinch test response scores described in the text are averaged over 2 raters for 3 pinch tests that followed each synchronous or mimicked stroking treatment conducted once per day for 5 total days per treatment in male and female mice.

|  |  | Synchronous | | | | |  | Visually mimicked | | | | |
| --- | --- | --- | --- | --- | --- | --- | --- | --- | --- | --- | --- | --- |
| Mouse | Sex | Day 1 | Day 2 | Day 3 | Day 4 | Day 5 |  | Day 1 | Day 2 | Day 3 | Day 4 | Day 5 |
| F1 | female | 0.333 | 0.250 | 0.250 | 0.417 | 0.000 |  | 0.167 | 0.250 | 0.083 | 0.250 | 0.000 |
| F2 | female | 0.250 | 0.750 | 0.250 | 0.417 | 0.333 |  | 0.250 | 0.167 | 0.167 | 0.417 | 0.083 |
| F3 | female | 0.000 | 0.750 | 0.083 | 0.000 | 0.000 |  | 0.000 | 0.250 | 0.000 | 0.000 | 0.000 |
| F4 | female | 0.167 | 0.500 | 0.167 | 0.667 | 0.000 |  | 0.083 | 0.083 | 0.083 | 0.083 | 0.000 |
| F5 | female | 0.833 | 0.250 | 0.250 | 0.333 | 0.417 |  | 0.250 | 0.417 | 0.083 | 0.083 | 0.000 |
| F6 | female | 0.417 | 0.500 | 0.250 | 0.250 | 0.167 |  | 0.333 | 0.250 | 0.167 | 0.250 | 0.250 |
| F8 | female | 0.083 | 0.000 | 0.167 | 0.167 | 0.167 |  | 0.250 | 0.250 | 0.167 | 0.250 | 0.083 |
| M1 | male | 0.250 | 0.000 | 0.000 | 0.000 | 0.583 |  | 0.083 | 0.000 | 0.000 | 0.167 | 0.000 |
| M2 | male | 0.083 | 0.000 | 0.167 | 0.167 | 0.000 |  | 0.083 | 0.083 | 0.000 | 0.000 | 0.000 |
| M3 | male | 0.000 | 0.667 | 0.667 | 0.000 | 0.000 |  | 0.000 | 0.000 | 0.000 | 0.000 | 0.500 |
| M4 | male | 0.083 | 0.000 | 0.417 | 0.167 | 0.000 |  | 0.333 | 0.000 | 0.083 | 0.000 | 0.250 |
| M5 | male | 0.000 | 0.167 | 0.583 | 0.667 | 0.000 |  | 0.000 | 0.167 | 0.000 | 0.083 | 0.333 |
| M6 | male | 0.500 | 0.500 | 0.750 | 0.083 | 0.250 |  | 0.333 | 0.167 | 0.083 | 0.000 | 0.500 |
| M7 | male | 0.167 | 0.167 | 0.000 | 0.333 | 0.500 |  | 0.250 | 0.333 | 0.000 | 0.250 | 0.000 |
| M8 | male | 0.083 | 0.583 | 0.000 | 0.000 | 0.167 |  | 0.083 | 0.083 | 0.083 | 0.000 | 0.167 |
